# Peripheral arterial disease: A high risk – but neglected – disease population

**DOI:** 10.1186/1471-2261-5-15

**Published:** 2005-06-22

**Authors:** Joseph Tomson, Gregory YH Lip

**Affiliations:** 1 University Department of Medicine, City Hospital, Birmingham B18 7QH, England, UK

## Abstract

Peripheral arterial disease (PAD) is a common, progressive manifestation of atherothrombotic vascular disease, which should be managed no different to cardiac disease. Indeed, there is growing evidence that PAD patients are a high risk group, although still relatively under-detected and under treated. This is despite the fact that PAD patients are an increased mortality rate comparable to those with pre-existing or established cardiovascular disease [myocardial infarction, stroke]. With a holistic approach to atherothrombotic vascular disease, our management of PAD can only get better.

Peripheral arterial disease (PAD) results from the narrowing of the blood vessels of the lower limbs, predominantly secondary to atherosclerotic vascular disease leading to compromised blood flow to the lower limbs. Risk factors associated with PAD include typical cardiovascular risk factors, such as older age, cigarette smoking, diabetes mellitus, hypercholesterolemia, and hypertension [1]. PAD is also very common in the western world – based on symptoms of intermittent claudication, PAD has a prevalence of up to 5%, but a higher prevalence is estimated in the general population [1,2]. As with much of cardiovascular disease, PAD can be progressive, which about a third report worsening symptoms and require surgical interventions over 5 to 10 years [3] as well as suffer a higher cardiovascular mortality risk [2-4]. One could argue that compared to the large publicity and public health initiatives on heart attacks and strokes, public recognition on the risks, morbidity and mortality associated with PAD has been relatively neglected.

Given that the pathology in PAD arises secondary to atherosclerosis (or atherothrombosis) affecting the vascular tree, PAD would be a very useful, subtle and early indicator of a high risk population. The realisation that the majority of those with PAD remain asymptomatic [4], and that they can be more effectively identified by simple non-invasive techniques warrants new hope for the primary prevention of cardiovascular risk.

In their recent article published in *BMC Cardiovascular Disorders*, Caro et al [5] embark upon estimating the burden of cardiovascular risk in terms of mortality, morbidity and associated risk factors in patients with PAD. Of note, they compared outcomes between the PAD cohort to those with reference populations with a first diagnosis of myocardial infarction and stroke. They report that the mortality rate amongst those with PAD was higher when compared to those who had an index myocardial infarction but lower than those who had suffered stroke. Importantly, the rates were comparable to the other high risk cohorts, with the crude five year death rate standing at 33.2% [26.6% for index myocardial infarction; 41.8% for index stroke]. Furthermore, at the end of the follow-up period, half of the patients with PAD were alive, compared to 59.6% of those in the myocardial infarction group and 51.3% of those with stroke, respectively[5]. Broadly similar findings have also been reported in previous studies involving patients with PAD symptoms [6,7].

As expected, risk factor analyses revealed that men with PAD were more likely to have atherothrombotic complications, angina, myocardial infarction, stroke, and death [5]. Also, the risk of myocardial infarction was significantly increased with in those aged over 65 years, especially with concomitant angina, diabetes mellitus, heart failure, and hypertension – the increase in cardiovascular risk in those with PAD was dependent on the number of additional risk factors at the time of diagnosis in comparison to those who did not have any other risk factors at baseline, illustrating how risk factors are additive to each other, resulting in a 'cumulative' cardiovascular risk[5]. The parallels with cardiac disease are uncanny.

The study by Caro et al [5] does merit some scrutiny. As it is a database/registry-type study, and there are limitations to the data presented. For instance, temporal changes in blood pressures and laboratory values are not available for further analyses, as well as detailed information on optimization of medical therapies. Data on concomitant risk factors such as atrial fibrillation [8] could have helped in further understanding their long term impact on PAD and outcome. Case ascertainment also remains an issue, as the PAD cohort in the study by Caro et al [5] was identified based on the ICD codes used in case records. Though different methods with varying complexities are used currently [9], the ankle brachial pressure index seems a simple effective and noninvasive solution and the best mode for diagnosis. However, it would be a daunting task to ensure confirmatory evidence in a standardized detailed population examination of this magnitude. One may argue that the patients with prior stroke and myocardial infarction were at a better advantage, making them a 'more scrutinized' cohort with better provisions for active secondary risk factor modification.

The conclusion that having PAD puts you at a much higher risk which is comparable to the reference populations is therefore a justified one, and a message that needs to be emphasized. Eventhough the majority of PAD subjects remain asymptomatic, they remain a high-risk population – probably even higher than those with single cardiovascular risk factors. Of concern, risk factor reduction by lipid modifying therapy and antiplatelet therapy in those with a diagnosis of PAD is less frequently applied [10] in comparison to those with cardiac disease. Thus, PAD patients may have had a less intensive risk reduction, leading to a high cardiovascular event rate.

Should we be surprised? Cynical cardiologists would argue that these aspects are well-known for heart disease, but as PAD commonly presents to vascular surgeons (at least in the UK) the onus is on them to initiate full cardiovascular risk prevention therapies (a 'best medical therapy' strategy), such as smoking cessation and the aggressive treatment of hypertension and lipids. However, many PAD patients also have cardiac problems, and may attend blood pressure and lipid clinics. Perhaps the time has come to organize vascular clinics jointly run by vascular surgeons and vascular physicians (even cardiologists!), as one possible solution. The approach to the PAD patient should not be simply surgery (or a stent) but should include focus on aggressive risk factor modification [11,12].

However, cardiac patients are blessed with many well conducted large randomized controlled clinical trials, which PAD patients often have to depend on subgroup analyses from these studies to inform their management. Though clinical trials of lipid-lowering therapy for patients with PAD per se are not available, a systematic review by the Cochrane collaboration of seven clinical trials [13] which included 698 patients with PAD, showed a substantial but not statistically significant reduction in total mortality (odds ratio 0.21; 95% confidence interval [CI], 0.03–1.17), with lipid-lowering therapy. Furthermore, there are new studies indicating atherosclerosis regression with aggressive lipid-lowering therapy and with antihypertensive therapy, offering an effective interventional target [14,15], again emphasising the role for aggressive risk factor modification. Antiplatelet therapy with aspirin or thienopyridine drugs (ticlopidine and clopidogrel) reduces the risk of cardiovascular events in patients with established atherosclerosis [16], but in PAD patients included in the Antithrombotic Trialists Collaboration metaanalysis, there was no significant difference in cardiovascular outcomes. As in cardiac disease, blockade of the renin-angiotensin system may offer another therapeutic target. For example, in the Heart Outcomes Prevention Evaluation (HOPE) study [17], 44% of the study population had PAD and an impressive reduction in cardiovascular events was seen in the ACE inhibitor (ramipril) group, the reduction being independent of blood pressure reduction.

In conclusion, Caro et al [5] allows us to take stock of our approach to managing PAD. PAD should be managed no different to cardiac disease. Indeed, there is now growing evidence that PAD patients are a high risk group, although still relatively under-detected and under treated. This is despite the fact that PAD patients are an increased mortality rate comparable to those with pre-existing or established cardiovascular disease [myocardial infarction, stroke]. With a holistic approach to atherothrombotic vascular disease, our management of PAD can only get better.

## Pre-publication history

The pre-publication history for this paper can be accessed here:


